# Shortness of breath and palpitations in an elderly man: Bad device behavior or malfunction?

**DOI:** 10.1002/joa3.12446

**Published:** 2020-10-17

**Authors:** Gregory P. Siroky, Shawn Lee, Ranjit Suri

**Affiliations:** ^1^ Mount Sinai Morningside Icahn School of Medicine at Mount Sinai New York NY USA

**Keywords:** atrioventricular dyssynchrony, elective replacement indicator, pacemaker syndrome, permanent pacemaker

## Abstract

70‐year‐old male with sinus node dysfunction (SND) and paroxysmal atrial fibrillation presents with shortness of breath and palpitations. Presenting EKG shows AF with rapid ventricular rates requiring direct current cardioversion (DCCV). Post‐DCCV EKG shows sinus rhythm with competing ventricular pacing. Device interrogation demonstrates the patient's generator at the elective replacement indicator (ERI) and has been forced to VVI 65 bpm causing dyssynchronous ventricular pacing and inducing AF. This case highlights the importance of close device follow up with timely PPM generator change prior to ERI, especially in patients with Medtronic Adapta devices, to avoid unnecessary dyssynchronous ventricular pacing. In addition, device manufacturers should focus on maintaining AV synchrony in pacemakers when they reach ERI.

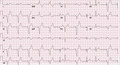

## 1 CASE

**FIGURE 1:**
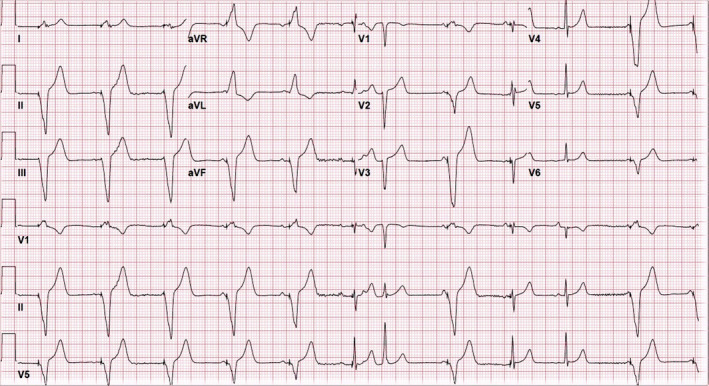
Postcardioversion ECG

A 70‐year‐old male presents with shortness of breath and palpitations. His history is remarkable for atrial fibrillation (AF) status postmultiple ablations and direct‐current cardioversions (DCCV) as well as sinus node dysfunction (SND) status postpermanent pacemaker (PPM) implant 12 years ago. Several days prior to presentation, he initially felt worsening shortness of breath and a day later developed severe palpitations. Upon admission, he was found to be in AF with rapid ventricular rate. He underwent successful DCCV with resultant ECG as shown in Figure 1. Unfortunately, the patient continued to experience dyspnea despite being free of palpitations. What is the interpretation of the ECG? Does the patient have a single or dual chamber permanent pacemaker (PPM)? What clues on the ECG allude to a problem with the PPM that requires fixing?

## 2 DISCUSSION

A step‐by‐step analysis of the ECG, as well as basic knowledge of PPM programming, is required to make the correct diagnosis. The ECG shows normal sinus rhythm at 68 bpm with competing ventricular demand pacing at 65 bpm. Scanning from left to right, the 1st, 2nd, 3rd, 4th, 5th, 8th, and 11th complexes are fully ventricular paced (pacing spike followed by a wide QRS complex). The 6th and 9th complexes are pseudofused (the pacemaker has already committed to pacing based on the set timing cycle, as seen by the pacing spike, despite native conduction resulting in a narrow QRS complex). The 7th complex is a result of a premature atrial contraction (PAC) (P wave can be seen just prior to the preceding T wave in the lead II rhythm strip). And finally, the 10th complex is a natively conducted sinus beat. With each QRS complex identified, we can now discuss whether this a single or dual chamber PPM. To do this, we must first determine if the native atrial and ventricular activity is being sensed appropriately. Just prior to the 4th ventricular paced complex, we can see a sinus P wave with a nonphysiologic PR interval just prior to ventricular pacing. This demonstrates that the PPM is programmed to ventricular pacing irrespective of atrial activity (VVI) which suggests two possibilities: (a) a single chamber ventricular demand (VVI) PPM, which, given the patient's history of SND, is possible but unlikely as the PPM was implanted in 2008 or (b) a dual chamber PPM set or mode switched to VVI only pacing. Therein lies the importance of being familiar with the idiosyncrasies of each individual pacemaker manufacturer. The rate of ventricular pacing and lack of atrial tracking/sensing is the clue to determine the PPM manufacturer as well as how and why the device is programmed. Based on the analysis of our patient's ECG, the PPM is programmed to VVI mode at a rate of 65 bpm. This mode and rate is specific only to Medtronic devices (Minneapolis, MN) when the PPM generator has reached its elective replacement indicator (ERI) and, unfortunately, does not maintain AV synchrony like other device manufacturers.^1^ In addition, once the generator has reached ERI, programming changes are not possible which, in our patient, was determined on interrogation prior to DCCV. There are an estimated 264 689 generators, specifically the older generation Adapta devices, implanted in patients with this feature.^2^ This issue has been previously reported and discussed with Medtronic as far back as 2010 with their response stating, “This mode and rate were seen as a safe compromise operation which provides a more predictable ERI to EOL (end‐of‐life) service life and an easily identifiable indicator via trans‐telephonic monitor or surface ECG/EKG strip.”^3^ In this day and age of remote monitoring, trans‐telephonic checks are obsolete and, as such, there should be improved methods to better surveil patients with the older generation Adapta devices before they reach ERI and are subjected to dyssynchronous pacing. Two newer generation Medtronic devices, Advisa (February 2013) and Azure (November 2017), have been released which still have the same ERI behavior as the Adapta, but now have a recommended replacement time (RRT) indicator which is activated 3 months prior to ERI and does not change any device programming.^2^ In addition, if the generator is allowed to reach ERI and automatically switches to VVI 65 bpm, reprogramming can be performed, however, the generator will reach EOL at a faster rate. Nonetheless, the impact of this feature on a patient's wellbeing can be quite troublesome leading to avoidable symptoms and unnecessary interventions, such as coronary angiograms and DCCV, as well as an overall increased cost to the healthcare system. When the generator is forced into a VVI 65 bpm mode at ERI, the pacemaker does not synchronously pace and leads to a profound version of pacemaker syndrome, causing symptoms such as shortness of breath, as occurred in our patient, and potentially chest pain. In addition, high ventricular pacing percentages, specifically right ventricular septal and apical pacing, have been shown to be associated with AF.^4^ Sweeney et al demonstrated that ventricular dyssynchrony as a result of ventricular pacing led to an increased risk of heart failure and AF in patients with SND and a normal QRS duration.^5^ When our patient's PPM reached ERI and automatically switched to VVI 65 bpm, this led to dyssynchronous ventricular pacing which competed with his underlying sinus rate of 68 bpm, ultimately causing his shortness of breath due to pacemaker syndrome. As a result of 100% ventricular pacing along with his history of AF, he converted to AF producing his symptom of palpitations requiring DCCV. He subsequently underwent PPM generator change and with restoration of normal dual chamber pacemaker function and has been free of symptoms. This case highlights the importance of close device follow‐up with timely PPM generator change prior to ERI, especially in patients with Medtronic Adapta devices, to avoid unnecessary dyssynchronous ventricular pacing. In addition, device manufacturers should focus on maintaining AV synchrony in pacemakers when they reach ERI.

## CONFLICT OF INTEREST

The authors declare no conflict of interest for this article.
